# Ultrasound-Based Recovery of Anti-Inflammatory and Antimicrobial Extracts of the Acidophilic Microalga *Coccomyxa onubensis*

**DOI:** 10.3390/md21090471

**Published:** 2023-08-27

**Authors:** Mari Carmen Ruiz-Domínguez, María Robles, Lidia Martín, Álvaro Beltrán, Riccardo Gava, María Cuaresma, Francisco Navarro, Carlos Vílchez

**Affiliations:** 1 Laboratorio de Microencapsulación de Compuestos Bioactivos (LAMICBA), Departamento de Ciencias de los Alimentos y Nutrición, Facultad de Ciencias de la Salud, Universidad de Antofagasta, Antofagasta 1240000, Chile; 2 Algal Biotechnology, CIDERTA-RENSMA, Faculty of Experimental Sciences, University of Huelva, 21007 Huelva, Spain; maria.robles@dqcm.uhu.es (M.R.); lidia.martin@dqcm.uhu.es (L.M.); maria.cuaresma@dqcm.uhu.es (M.C.); cvilchez@dqcm.uhu.es (C.V.); 3 Bioplagen S.L., Av. Castilleja de la Cuesta, 20-22, Bollullos de la Mitación, 41110 Seville, Spain; alvarobeltran@bioplagen.com (Á.B.); riccardogava@bioplagen.com (R.G.); 4 Cell Alterations by Exogenous Agents, RENSMA, Department of Integrated Sciences, Faculty of Experimental Sciences, University of Huelva, 21007 Huelva, Spain; fnavarro@uhu.es

**Keywords:** *Coccomyxa onubensis*, ultrasound-assisted extraction, lutein recovery, antimicrobial activity, sterols, food applications

## Abstract

In the present study, the recovery of valuable molecules of proven anti-inflammatory and antimicrobial activity of the acidophilic microalga *Coccomyxa onubensis* (*C. onubensis*) were evaluated using green technologies based on ultrasound-assisted extraction (UAE). Using a factorial design (3 × 2) based on response surface methodology and Pareto charts, two types of ultrasonic equipment (bath and probe) were evaluated to recover valuable compounds, including the major terpenoid of *C. onubensis*, lutein, and the antimicrobial activity of the microalgal extracts obtained under optimal ultrasound conditions (desirability function) was evaluated versus conventional extraction. Significant differences in lutein recovery were observed between ultrasonic bath and ultrasonic probe and conventional extraction. Furthermore, the antimicrobial activity displayed by *C. onubensis* UAE-based extracts was greater than that obtained in solvent-based extracts, highlighting the effects of the extracts against pathogens such as *Enterococcus hirae* and *Bacillus subtilis*, followed by *Staphylococcus aureus* and *Escherichia coli*. In addition, gas chromatography–mass spectrometry was performed to detect valuable anti-inflammatory and antimicrobial biomolecules present in the optimal *C. onubensis* extracts, which revealed that phytol, sterol-like, terpenoid, and even fatty acid structures could also be responsible for the antibacterial activities of the extracts. Moreover, UAE displayed a positive effect on the recovery of valuable molecules, improving biocidal effects. Our study results facilitate the use of green technology as a good tool in algal bioprocess engineering, improving energy consumption and minimizing environmental impacts and process costs, as well as provide a valuable product for applications in the field of biotechnology.

## 1. Introduction

Microalgae are a diverse group of unicellular photosynthetic microorganisms found in various aquatic habitats, including marine, freshwater, and brackish water environments [1,2]. They are one of the most promising biomass sources for biotechnological applications owing to their high growth rates, ability to produce various valuable compounds, and low environmental impact. In particular, extremophile microalgae offer numerous biotechnological benefits owing to their unique ability to adapt to extreme environments. These microorganisms have garnered considerable attention because of their potential applications in various industries, including food, pharmaceutical, and cosmetics, owing to their ability to produce pigments, antioxidants, and other bioactive molecules [3,4]. *Coccomyxa onubensis* is an acidophilic microalga and a well-known producer of the proven anti-inflammatory and antimicrobial terpenoid lutein [5,6,7]. It exerts antibacterial activity [8] and is considered a safe food source for animals, improving the antihyperglycemic and antihyperlipidemic protective effects on rats [9,10]. This microalga was isolated from the acidic mine drainages of the Pyritic Belt located southwest of Andalusia (Huelva, Spain), an environment suitable for the growth of many extremophile microorganisms; this area has high concentrations of heavy metals, low pH, and is exposed to a high UV light irradiation [6].

Microalgae have been explored for their antimicrobial activities, which have been attributed to different chemical compounds, including indoles, terpenes, acetogenins, phenols, fatty acids, and volatile halogenated hydrocarbons [11]; most of these compounds have been also attributed anti-inflammatory properties through in vitro assays [12,13]. In general, to obtain high-value products, the extraction technique is an important step in algal bioprocess engineering. The selection of the extraction technique can substantially affect the energy consumption of the overall production process. A suitable extraction technique minimizes environmental effects and associated process costs and increases product value [14].

Ultrasound-assisted extraction (UAE) is a green extraction technology primarily based on the cavitation phenomenon [15]. The thermal and mechanical effects of ultrasound waves on the medium, trigger biomass rupture [16]. These characteristics of UAE significantly improve the mixing and high mass transfer of the solvent into the sample matrix and establish a greater surface contact area between the solid and liquid phases [17]. Two types of ultrasonic equipment are commonly used to perform UAE: probes (high ultrasound intensity) and baths (high ultrasound intensity) [18]. These aspects lead to considerable differences in the recovery of valuable molecules from the extraction processes [18,19]. Several studies have reported the use of ultrasound technology for carotenoid recovery from algal biomass using different ultrasound frequencies, from low frequencies of 18–200 kHz to high frequencies of 400–10 MHz [15,20]. However, only a few studies have described the effects of this technology on the antimicrobial activity of the resulting microalgae extracts [21,22]. The novelty of this study is not related to the use of UAE technologies but to their specific application to developing green extraction processes from acidophilic microalgae; extremophilic microalgae are gaining relevance in biotechnology [5,6,10], and extraction procedures must be developed which unveil novel active molecules and efficient recovery protocols.

Therefore, we studied the effects of UAE on lutein extraction yield and other valuable metabolites in *C. onubensis* extracts that can exert antimicrobial and anti-inflammatory activity; the former activity was determined in the extracts, and the latter activity was proposed based on the unveiled presence of specific compounds having been reported to exert anti-inflammatory activity. This study was performed by using two types of ultrasonic equipment: an ultrasonic bath and probe (50 Hz and 20 kHz of ultrasound frequency, respectively), and the results were compared with those obtained by conventional extraction using the non-green solvent methanol (maceration). All experiments were performed using factorial designs (3 × 2) based on response surface methodology (RSM) and Pareto charts, using ethanol as the green extractant.

## 2. Results and Discussion

### 2.1. Effects of the Ultrasonic Techniques on the Extraction Yield of C. onubensis

Ultrasonic techniques are known for improving the recovery of valuable molecules compared with other methods and have been tested for biomass of many origins such as fruits and vegetables, algae, or agri-food wastes among other sources [21,23]. In the present study, we elucidated the differences between an ultrasonic bath (Table 1) and an ultrasonic probe (Table 2) under two different factorial designs (3 × 2) to determine the effects of two factors with three levels in eleven runs (with two additional central points) on the extraction yield and recovery of the anti-inflammatory and antimicrobial terpenoid lutein, used for evaluating the recovery method efficiency of proven anti-inflammatory and antimicrobial lipophilic compounds of the acidophilic microalga *C. onubensis* extracted with ethanol (a type of green solvent).

**Figure 1:**
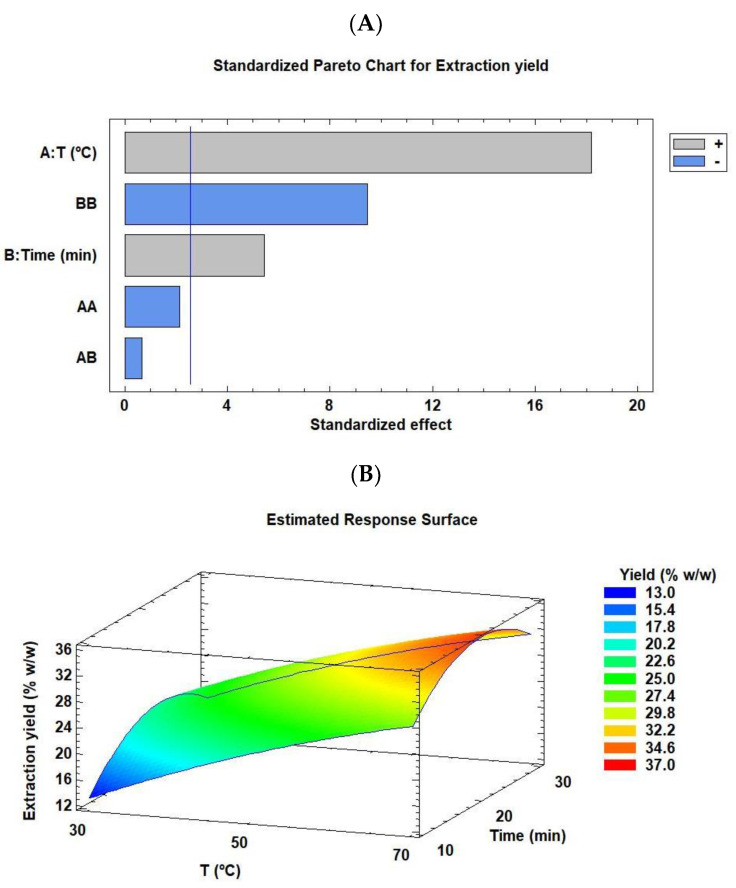
Pareto chart (**A**) and RSM (**B**) rationalizing the effect of each factor on the extraction yield of *C. onubensis,* using an ultrasonic bath. The vertical line in the Pareto chart indicates the 95% confidence level for the effects. Note: RSM, response surface methodology. The factors were extraction time (10, 20, and 30 min) and temperature (30 °C, 50 °C, and 70 °C).

The factors for the ultrasonic bath were extraction time (10, 20, and 30 min) and temperature (30 °C, 50 °C, and 70 °C). Table 1 indicates that the optimal condition for maximum extraction yield was run 6, i.e., extraction at 70 °C for 20 min, with an extraction yield of 35.15% *w*/*w*, followed by runs 9 and 5 (yields of 31.48% and 31.27% *w*/*w*, respectively). However, the extraction conditions of runs 1 and 7 (low temperature of 30 °C for low-middle extraction time of 10 and 20 min, respectively) obtained the poorest extraction yield of 13.05% and 17.82% *w*/*w*, respectively. Figure 1 illustrates the Pareto chart (Figure 1A) and RSM plot (Figure 1B) of *C. onubensis* after adjusting the effect of each factor (extraction time and temperature) on the extraction yield using the ultrasonic bath. The Pareto chart (Figure 1A) revealed that factors such as temperature, extraction time, and quadratic extraction time were statistically significant in the extraction (*p* ≤ 0.05). In particular, temperature and extraction time improved the process under high values (Table 1, runs 6 and 9), whereas quadratic extraction time provided opposite results. On the other hand, the statistical software complemented with the complete mathematical model (Equation (1)), with an *R*^2^ value of 98.96%; this indicates that this model has a good fit (Table S1). In the equation given below, the extraction yield was represented using T as the extraction temperature (In °C) and t as the extraction time (In min) and their combinations.

Yield = 29.5221 + 7.09833 × T + 2.12833 × t − 1.29026 × T^2^ − 0.3175T × t − 5.69026 × t^2^ (1)

The nonsignificant terms from the model were excluded and the mathematical model was refitted to obtain a new equation (Equation (2)) with a similar deviation (*R*^2^ = 97.90%).

Yield = 29.006 + 7.09833 × T + 2.12833 × t − 6.03433 × t^2^ (2)

Furthermore, Figure 1B (RSM) illustrates that the color intensity increased with extraction at high temperatures and moderately high extraction times, as explained above. The optimal conditions obtained using the statistical software were approximately 70 °C and 22 min (Supplementary Materials, Table S1), with an optimal extraction yield of 36.29% *w*/*w*, which is extremely close to the experimental data (run 6).

Table 2 presents the extraction yield data of *C. onubensis* after using an ultrasonic probe. In this experiment, the factors were pulse duration and pulse interval (including varying sonication times of ON equal to 10 s and OFF from 30 to 60 s) and extraction time (5, 10, and 15 min).

**Figure 2:**
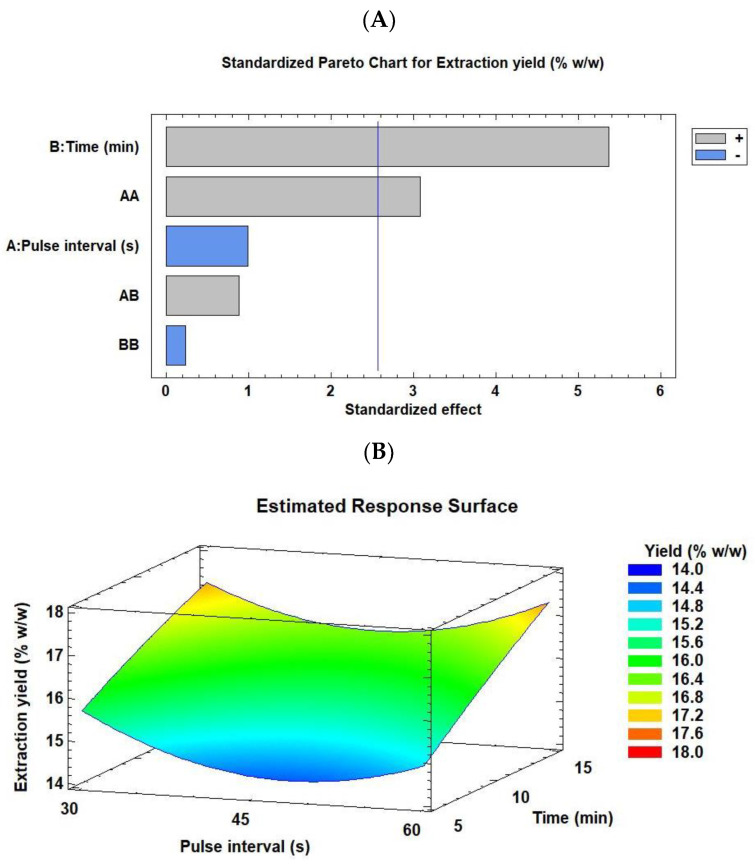
Pareto chart (**A**) and RSM (**B**) rationalizing the effect of each factor on the extraction yield of C. onubensis obtained using an ultrasonic probe. The vertical line in the Pareto chart indicates the 95% confidence level for the effects. Note: RSM, response surface methodology. Pulse intervals understanding as the “off” ultrasonic time (“on mode” was 10 s in all cases). The factors were pulse interval (10/30, 10/45, and 10/60 s/s) and extraction time (5, 10, and 15 min).

In general, the extraction yield data of the probe were lower than those obtained using an ultrasonic bath. The best yield was 17.34% *w*/*w* (run 9), closely followed by 17.17% *w*/*w* (run 4) (Table 2). In both cases, the extraction conditions were opposite, with run 9 being a high-level condition (10/60 s and 15 min) and run 4 being a low-middle level condition (10/30 s and 10 min). The worst extraction yield obtained was not very low (14.57% *w*/*w*) and was observed for run 2 (10/45 s and 5 min). Figure 2 summarizes the Pareto chart (Figure 2A) and RSM plot (Figure 2B) for the extraction yield of *C. onubensis* obtained using an ultrasonic probe. Extraction time and quadratic pulse interval were factors significantly and positively affecting the extraction process. In addition, a model equation (Equation (3)) was calculated for yield under the ultrasonic probe condition using statistical software (*R*^2^ = 88.98%, Table S2).

Yield = 15.4821 − 0.188333 × Pi + 1.01833 × t + 0.899737 × Pi^2^ + 0.205 Pi × t − 0.0702632 × t^2^ (3)

where Pi indicates the pulse intervals (s/s), and t indicates the extraction time (min). The nonsignificant terms of the initial model (pulse, quadratic extraction time, and their interactions) were excluded and the following equation was obtained (Equation (4)) with an *R*^2^ of 84.97%.

Yield = 15.454 + 1.01833 × t + 0.881 × Pi^2^ (4)

As demonstrated in the RSM plot (Figure 2B), a high extraction time (15 min) and extreme pulse intervals (10/30 and 10/60 s/s) improved the extraction yield of *C. onubensis* extracts using an ultrasonic probe. For the optimal extraction conditions predicted using the statistical software for yield, the maximum yield was obtained at 10/60 s/s and 15 min, with a projected value of 17.35% *w*/*w*.

Studies have recommended using ultrasound as a pretreatment process for cell disruption in the microalgal biorefinery process because it confers advantages such as high efficiency, mild operating conditions, low toxicity, and time-saving methodology [24,25]. We observed that both ultrasound techniques increased the extraction yield of *C. onubensis* in lower extraction times, resulting in up to 2.9- and 1.4-fold higher yields for the ultrasonic bath and probe, respectively, compared with maceration (12.31% ± 0.02% *w*/*w*). Therefore, compared with conventional extraction, UAE allows greater permeability of the biomass with the ethanol solvent to recover the biomolecules present in *C. onubensis*.

Regarding the specific ultrasonic methods used in this work, ultrasonic bath improved the extraction yield compared with ultrasonic probe. This could be because of the formation and accumulation of radicals during the cavitation process under high ultrasound intensity (the frequency was 20 kHz or 226 W/cm^2^ of acoustic power delivered into a liquid for the ultrasonic probe versus 50 Hz for the ultrasonic bath). Pingret et al. [26] comprehensively described the physicochemical effects of UAE in food processing. Acoustic cavitation is characterized by an increase in temperature and pressure conditions. It confers beneficial effects on the extraction of bioactive compounds; however, it can also alter the extraction conditions by producing radicals and molecules such as OH and H radicals, resulting in substantial quality defects in these products. Vintila et al. [27] also confirmed similar results using an ultrasonic bath. They increased the ultrasound intensity from 60% to 100% and demonstrated that the extraction yield of carotenoids and lipids considerably decreased. Therefore, the effects of ultrasound intensity or power input on the extractability of a target component are complex and warrant additional studies into the detailed extraction procedure.

### 2.2. Effects of the Ultrasonic Techniques on the Anti-Inflammatory Carotenoids Profile of C. onubensis

**Figure 3:**
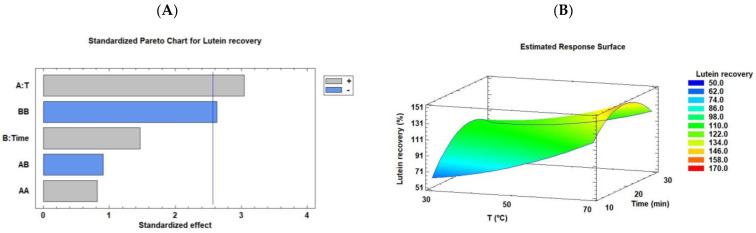
Pareto chart (**A**) and RSM (**B**) rationalizing the effect of each factor on lutein recovery from *C. onubensis*, using an ultrasonic bath. The vertical line in the Pareto chart indicates the 95% confidence level for the effects. Note: RSM, response surface methodology.

Lutein is the main anti-inflammatory carotenoid present in *C. onubensis* (~70% of the total carotenoids quantified). Figure S1 illustrates the HPLC profile, which demonstrated that other major, anti-inflammatory carotenoids such as neoxanthin, violaxanthin, zeaxanthin, astaxanthin, and β-carotene are present in lower contents in this microalga. Studies have reported that *C. onubensis* accumulates high levels of lutein, which is improved by cultivating the microalgal cultures under different conditions, modifying the lutein synthesis route as an antioxidant protector [6,7,28]. We evaluated the effects of the two ultrasonic modes (bath and probe) on lutein recovery from *C. onubensis* using an experimental design (3 × 2). Lutein recovery represents the achieved percentage of lutein using UAE compared with conventional extraction (3.24 mg/g of lutein with respect to biomass grams, benchmark extraction). Table 1 suggests that the best conditions for lutein recovery using an ultrasonic bath were runs 9 and 6 (70 °C/30 min and 70 °C/20 min, respectively), with recoveries of 134.27% and 129.93% *w*/*w*, respectively. However, the condition of low temperature and extraction time (30 °C/10 min, run 1) was noted to be the worst for lutein recovery (51.81% *w*/*w*). These data are complemented by the data illustrated in Figure 3, which presents the Pareto chart (Figure 3A) and RSM plot (Figure 3B) of lutein recovery from *C. onubensis* using an ultrasonic bath. The positively significant factor was temperature, followed by quadratic extraction time (*p* ≤ 0.05). The optimal response obtained using the statistical software was at 70 °C for 20.43 min (148.96% *w*/*w*). In general, compared with conventional extraction (over 100% of lutein recovery), this ultrasonic technique improved lutein extraction. The completed regression equation (Equation (5)) was fitted to the data as follows:

Lutein recovery = 119.818 + 20.5667 × T + 9.88333 × t + 8.51895 × T^2^ − 7.505 × T × t − 27.3511 × t^2^ (5)

where T is the extraction temperature (in °C), and t is the extraction time (in min). The *R*^2^ value was 79.34% (Table S1). Equation (6) shows the mathematical model with the significant factors of the initial model (extraction temperature and quadratic extraction time). Other nonsignificant variables were excluded, and the following equation was obtained (Equation (6)) with an *R*^2^ of 64.29%:

Lutein recovery = 123.226 + 20.5667 × T − 25.0793 × t^2^ (6)

RSM (Figure 3B) was used to simultaneously optimize the levels of these variables to obtain the system with the best performance based on the fit of a polynomial equation to the experimental data [29]. In this case, the plot corresponded to the results described above, where the optimal condition was high extraction temperature (70 °C) and moderate extraction time (~20 min).

**Figure 4:**
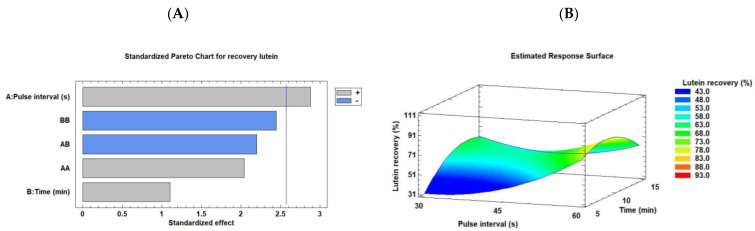
Pareto chart (**A**) and RSM (**B**) rationalizing the effect of each factor on the lutein recovery from *C. onubensis,* using an ultrasonic probe. The vertical line in the Pareto chart indicates the 95% confidence level for the effects. Note: RSM, response surface methodology. Pulse interval understanding as the “off” ultrasonic time (the “on” mode was 10 s in all cases).

Next, we elucidated lutein recovery from *C. onubensis* using an ultrasonic probe. As shown in Table 2, the data ranged from 37.85% to 97.85% *w*/*w*. These values were lower than those obtained using the ultrasonic bath. Nevertheless, the best extraction condition was observed for run 6 (1/60 s/s of pulse and 10 min, 97.86% *w*/*w*), followed by run 3 (1/60 s/s of pulse and 5 min, 69.88% *w*/*w*). Figure 4A,B illustrate the Pareto chart and RSM plot, respectively. The Pareto chart revealed that pulse interval was the unique significant variable in the lutein extraction from *C. onubensis,* calculated as lutein recovery. This factor was defined as the “off” ultrasonic time (30, 45, and 60 s). The “on” mode was 10 s in all cases. By increasing the pulse interval (10/60 s), lutein recovery also improved. However, extraction time (in min) was a nonsignificant variable as well as the combination or quadratic of these factors. Figure 4B (RSM) demonstrates that intense color was observed under elevated pulse intervals (in s) and intermediate extraction time conditions. The optimal condition obtained using the software was a pulse of 10/60 s/s for 9 min, with an optimal recovery of 84.77% *w*/*w*. The regression equation (Equation (7)) was fitted to the data to obtain the mathematical model as follows:

Lutein recovery = 60.4063 + 11.335 × Pi + 4.34333 × t + 12.3692 × Pi^2^ − 10.615 × Pi × t − 14.8358 × t^2^ (7)

where the values of the variables are specified in their original units (Pi, pulse intervals in s/s and t, extraction time in min) and *R*^2^ was 81.73% (Table S2). To simplify the obtained mathematical model, the nonsignificant variables in the lutein recovery process were excluded. As a result, the following equation (Equation (8)) with a very low *R*^2^ of 30.21% *w*/*w* was obtained.

Lutein recovery = 59.0609 + 11.335 × Pi(8)

In general, the effect of ultrasound intensity on the extractability of a target component from different natural sources is complex and warrants further investigation into the detailed extraction procedure. In the present study, the lutein recovery data of *C. onubensis* using an ultrasonic bath were higher than those using an ultrasonic probe. This could be because of the high ultrasound intensity used in the sonicator (50 kHz), resulting in the damaging or degrading effect of ultrasonic waves on the pigments. In fact, in the ultrasonic probe, when the pulsed interval was longer (10/30 s/s for 5 min vs. 10/60 s/s for 10 min), the lutein recovery increased from 37.85% to 97.85% *w*/*w*, respectively. On the other hand, for the ultrasonic bath, which used a less intense ultrasound frequency (50 Hz) but high temperatures (30–70 °C), lutein recovery was 1.4-fold higher than the best result of the ultrasonic probe (run 6, Table 2). Extraction temperature can be another relevant factor in the extraction process. In the ultrasonic probe experiment, the temperature was controlled at 12 °C ± 4 °C to avoid the degradation of thermosensitive bioactive compounds and formation of vapor-filled bubbles (cushioning effect) [30]. Although the ultrasonic bath had a lower ultrasound intensity than the ultrasonic probe, the increase in temperature significantly affected the performance of the solvent by improving its diffusion rate and mass transfer capacity with the sample.

Several studies have described the importance of temperature in UAE and have highlighted that low temperatures (<30 °C) can exert a beneficial effect on the extraction process; in contrast, temperatures above 75 °C may increase the degradation of the obtained compounds [31,32]. As a result, the processing temperature should be optimized to obtain the highest extraction yield.

In a previous study, supercritical CO_2_ extraction, another green extraction technique, was performed to optimize the extraction of valuable molecules in *C. onubensis* [33]. The lutein recovery value was less than that obtained using UAE in the present study (up to 50% of that of the optimal ultrasound bath condition). Similar findings were obtained for extraction yield, with 2-fold more yield under the bath condition. Deenu et al. [34] used RSM to optimize UAE experiments with or without enzymatic pretreatment to obtain the optimal conditions for lutein extraction from the microalga *Chlorella vulgaris*. They revealed that the optimal lutein recovery was 3.16 ± 0.03 mg/g wet weight of *Chlorella vulgaris* under the following UAE conditions: frequency, 35 kHz; ultrasound intensity, 56.58 W/cm^2^; extraction temperature, 37.7 °C; extraction time, 5 h; and solvent–biomass ratio, 31 mL/g. This value was for the experiment without enzymatic pretreatment, which was similar to that with enzymatic pretreatment (3.36 mg/g wet weight of *Chlorella vulgaris*). Regarding the best lutein yield calculated using dry biomass, the optimal value was 12.38 mg/g dry weight of *Chlorella vulgaris* (approximately three times more than the optimal lutein content of *C. onubensis* using an ultrasonic bath, run 9). Although extraction was performed for 5 h in the previous study and for 15–30 min in the present study, studies can be performed to modify extraction times.

Another important concept is the stability of the bioactive compounds extracted using UAE. Sun et al. [35] investigated the effects of different UAE factors on the stability of all *trans*-β-carotene in a model system and the degradation kinetics and products. They varied the intensities from 5% to 85% (corresponding to 60.5 and 1028.9 W/cm^2^) for 10 min on pulsed mode (2 s on and 2 s off) with temperature control. Their findings were consistent with those of our study; the concentration of β-carotene in dichloromethane decreased to approximately 70% when ultrasound intensity was varied from 60.5 to 302.5 W/cm^2^.

Notably, culture conditions also play an important role in increasing lutein production in microalgae [36]. In summary, UAE improves lutein recovery from *C. onubensis* and can be a sustainable green process that can be applied in extreme microalgae biorefineries without overlooking that ultrasound power intensity is a critical parameter that requires optimization.

### 2.3. Desirability Function

**Figure 5:**
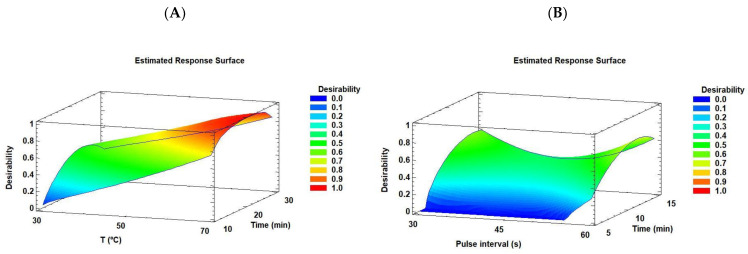
Graphical representation of the desirability functions for the different optimization criteria for (**A**) ultrasonic bath and (**B**) ultrasonic probe.

The desirability function approach is one of the most widely used methods for optimizing multiple response processes. In the present study, it was based on maximizing variable responses such as extraction yield and lutein recovery. Using this approach, we identified the specific operating conditions that provide the “most desirable” response values from the UAE of *C. onubensis*. For the ultrasonic bath, the optimal conditions were an extraction temperature of ~70 °C and time of ~22 min (optimum value = 1.0, extraction yield of 35.33% *w*/*w* and lutein recovery of 147.34% *w*/*w*). For the ultrasonic probe, the factors were an extraction time of ~12 min and pulsed duration/interval of ~10/60 s of the sonicator (optimum value = 0.736, extraction yield of 16.8% *w*/*w* and lutein recovery of 78.7% *w*/*w*). The biomass–solvent ratio was the same (1:100) in all experiments. Figure 5A,B illustrate the desirability graphs for the ultrasonic bath and probe. In these optimized techniques, both responses (extraction yield and lutein recovery) were transformed into a dimensionless individual desirability function ranging from 0 to 1, with 0 corresponding to the lowest desirability level and 1 to the most desirable condition. Good agreement was observed between the predicted and experimental responses at the optimal conditions, with run 6 being the optimal response for the ultrasound bath and probe experiments (Table 1 and Table 2). Taken together, these results suggest that the ultrasonic bath equipment used in this study exhibits good performance for the extraction yield and lutein recovery of *Coccomyxa onubensis* because this green technology positively contributed to both responses.

Subsequently, the predicted conditions obtained using the desirability function were used to evaluate the antimicrobial effects of *C. onubensis* extracts. The ethanolic aliquots were evaporated under a nitrogen stream and resuspended in DMSO. The methanolic extract was obtained using the conventional extraction method (solvent extraction).

### 2.4. Antimicrobial Activity and Anti-Inflammatory Metabolite Identification

Table 3 presents the antimicrobial activity of *C. onubensis* extracted using UAE and conventional extraction against known Gram-negative and Gram-positive bacteria: *Pseudomonas aeruginosa*, *Escherichia coli*, *Staphylococcus aureus*, *Enterococcus hirae*, and *Bacillus subtilis*. The assay was performed in vitro using the broth microdilution method, one of the most used methods to determine the MIC of antimicrobial agents, including antibiotics and other substances that can kill (bactericidal activity) or inhibit the growth (bacteriostatic activity) of bacteria. The methods described here are targeted for testing the susceptibility to antibiotic agents, rather than other antimicrobial biocides such as preservatives and disinfectants. Serial dilutions of *C. onubensis* extracts, ranging from 0.50 µg/mL to 2.20 mg/mL, were assayed.

In all extraction methods using ethanol or methanol as the extractant, relatively low concentrations of the extracts exhibited high efficiency against all pathogens (Table 3).The authors of [37] described the ratings of the antimicrobial efficiency of extracts based on their MIC values as follows: strong inhibitor, MIC < 500 µg/mL; moderate inhibitor, MIC of 600–1500 µg/mL; and weak inhibitor, MIC > 1600 µg/mL. Based on these criteria, *C. onubensis* extracts exhibited strong inhibition against selected pathogens. In particular, UAE (ultrasonic bath and probe) improved the antimicrobial activity of the extracts against *Enterococcus hirae* and *Bacillus subtilis*, followed by *Staphylococcus aureus* and *Escherichia coli* (MIC data range of 2–276 µg/mL of extracts). The antimicrobial activity of the conventional extract against *Pseudomonas aeruginosa* was better than that of *C. onubensis* extracted using UAE. In addition, *C. onubensis* extracts were more effective against Gram-positive bacteria than against Gram-negative bacteria. This effect has been previously described in antibiotics because Gram-positive bacteria have a complex and multilayered cell wall, making it difficult for the active compounds to penetrate the bacteria [38]. The biocidal effects of *C. onubensis* extracts were higher than those of extracts of other microalgae species. Saeed Niazi et al. [39] investigated the antimicrobial potential of the methanol extracts of the green microalgae isolated from the Persian Gulf and highlighted their effects against *Staphylococcus aureus*, *Bacillus Cereus*, *Escherichia coli*, and *Pseudomonas aeruginosa* (the MIC was 0.75, 1.5, 3, and 6 mg/mL and MBC was 1.5, 1.5, and 6 mg/mL, respectively).

The aim of the broth dilution method is to determine the lowest concentration of the assayed antimicrobial agent (MIC) that, under defined test conditions, inhibits visible bacterial growth. MIC values are used to determine the susceptibilities of bacteria to drugs. Furthermore, they are used to evaluate the activity of new antimicrobial agents. In the broth dilution method, often determined in the 96-well microtiter plate format, bacteria are inoculated into a liquid growth medium in the presence of different concentrations of the antimicrobial agent. Growth is assessed after incubation for a defined period (16–20 h) and the MIC value is determined. However, this method only applies to aerobic bacteria and can be completed in 3 days [40]. Notably, DMSO at concentrations of 15% or lower does not affect the growth of the microorganisms tested, as indicated by Navarro et al. [8].

Solvent selection is vital for determining the antimicrobial compounds (including various chemicals) derived from microalgae [41]. For example, a polar solvent such as ethanol (dielectric constant of 24.3) will mainly extract polar compounds such as polar pigments and phenolic compounds; it is widely used owing to its low toxicity and high extraction yields [42].

To identify the compounds present in the most effective antimicrobial extracts (namely, conventional, ultrasonic bath, and ultrasonic probe), GC–MS analysis was performed. Among the extracted compounds, those having been reported to exert antimicrobial and anti-inflammatory properties were identified, though anti-inflammatory activity of the extracts was not analyzed in this study. Table 4 lists the masses obtained, which were compared with the exact masses from different libraries using the NIST MS 2.3 software, to identify the potential compounds and based on the antimicrobial activity in the literature.

Independently of the extraction method used, there were no differences in the type of compounds identified. As per GC–MS analysis, a common qualitative pattern was observed in the major biomolecular structures found in the extracts, which included phytol residues, fatty acid residues, sterol, and terpenoid compounds. Table 5 lists the name and properties of the antimicrobial molecules present in the microalgal extracts and detected via GC–MS analysis.

The aim of the antimicrobial activity assay was not to detect novel, natural antibiotic compounds. We believe that the path from detecting antibiotic compounds in natural extracts to their formulation as an antibiotic drug for humans is complex; moreover, a potentially useful natural antibiotic should, for instance, display an extremely high specific antibiotic activity against a resistant, pathogenic microorganism in humans. This will probably awaken the interest in the identified molecule as a potential commercial drug. However, the cost of producing microalgal biomass enriched in a given molecule and purifying it until homogeneity remains considerably higher than the production costs of common chemicals with antibiotic properties. Indeed, a molecule such as lutein, for example, can be accumulated by up to several milligrams per biomass gram of *C. onubensis*. Assuming 1% (dry weight basis) of lutein accumulation in the acidophilic microalgal biomass, 1 kg of biomass would be required to produce 1 g of lutein. The cost for producing 1 kg of biomass can approximately be even more than 10€, depending on the cultivation conditions and procedure. Furthermore, the compound must be extracted and purified to formulate it as a drug for the healthcare industry. Therefore, we suggest using biomass extracts enriched in bioactive compounds, such as anti-inflammatory and antimicrobial bioactive molecules, as a more feasible strategy to formulate products that can have further application in several daily human activities in different fields, including disinfection or nutraceuticals promoting healthy body’s normal state, rather than producing natural large molecules as nonspecific anti-inflammatory or antibiotic drugs.

**Figure 6:**
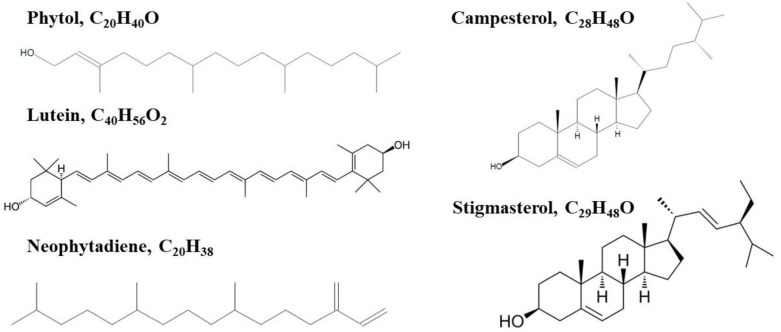
Chemical structures of the compounds displaying antimicrobial activity identified in alcohol solvent-based *C. onubensis* extracts.

A small number of structures that can be responsible for a part of the antimicrobial activity were identified in *C. onubensis* extracts. Phytol was one such structure. It is a diterpene alcohol (Figure 6) and a natural compound that chemically belongs to the class of diterpenes. Furthermore, phytol is part of the chemical structure of the major photosynthetic pigment chlorophyll and can be extracted using ethanol or methanol, among other solvents. Phytol exhibits weak anti-inflammatory and antimicrobial properties; however, it can be converted to other molecules exhibiting antimicrobial properties. Some prominent examples are phytol-derived compounds such as phytol acetate and phytol esters; these compounds possess antimicrobial properties against specific bacterial strains, including *Staphylococcus aureus* and *Escherichia coli*. The stronger antimicrobial properties of phytol derivates can increase their potential as preservative components in antimicrobial coatings and packaging materials.

Lutein (Figure 6) is a naturally occurring carotenoid pigment found in microalgae, with *C. onubensis* being an outstanding example of a potential producer. Recent studies have revealed its potential anti-inflammatory and antimicrobial properties [44,45]. Furthermore, several studies have reported that lutein exhibits antimicrobial activity against many microorganisms. In particular, lutein inhibits the growth of pathogenic bacteria such as *Staphylococcus aureus* and *Escherichia coli* [45]. Although the mechanisms of action of lutein as an antimicrobial compound remain unelucidated, lutein can interfere with bacterial cell membrane integrity; this is coherent with the linear, long-chain hydrocarbon nature of its chemical structure. The antimicrobial properties of lutein are attributed to its antioxidant and anti-inflammatory effects. Lutein can scavenge reactive oxygen species and decrease oxidative stress, a cellular process frequently associated with microbial infections. Furthermore, its anti-inflammatory properties help to modulate immune responses and contribute to its antimicrobial effects. Neophytadiene is a diterpene (Figure 6) that exerts antimicrobial, antioxidant, anti-inflammatory, and anxiolytic-like activities. However, only a few studies have reported the presence of neophytadiene in microalgae, with no study on its presence in acidophilic microalgae. Neophytadiene has been identified in plants, particularly in conifers. Nevertheless, their recently unveiled pharmacological properties may increase the interest in specific microalgae species as bioresources with the potential for producing neophytadiene. Although the biological activities of neophytadiene have not been extensively studied, it plays a vital role in cell defense mechanisms against oxidative stress. Stigmasterol and campesterol, sterol-derived structures (Figure 6), have been identified in *C. onubensis* extracts. Stigmasterol was originally identified as a typical sterol in fungi; however, it has also been identified in certain microalgae species, including *Chlorella*, *Nannochloropsis*, *Dunaliella*, and *C. onubensis* (the present study). Campesterol is a phytosterol typically found in fruits and vegetables. Nevertheless, the specific biological functions of both sterols in microalgae remain unelucidated. However, their potential roles in membrane fluidity regulation and stress response mechanisms, including UV light and extreme temperature, have been reported [46].

In microalgae, the abovementioned compounds, whose structures are all shown in Figure 6, are biochemically synthesized via the non-mevalonate sterol biosynthesis pathway, which is also called the 1-deoxy-D-xylulose-5-phosphate/2-methyl-D-erythritol-4-phosphate pathway. In microalgae, this pathway is activated under different stress conditions and meets the common features of inducing oxidative stress: high-light irradiance, UV radiation, extreme temperature, or presence of metal ions (a typical chemical scenario in the highly acidic, natural habitat of *C. onubensis*). However, this pathway does not create such oxidative scenarios in microalgal cultures [47,48].

## 3. Materials and Methods

### 3.1. Biomass and Chemicals

The microalga used in this study was *C. onubensis* (SAG 2510). The biomass was kindly donated by the research group Algal Biotechnology from the University of Huelva (Spain) and was previously described by Ruiz-Domínguez et al. [33]. The main chemical compound used in UAE was ethanol (99.5%) as a green solvent; it was purchased from VWR Prolabo Chemicals (Barcelona, Spain). Other chemicals used in chromatographic analyses were ethyl acetate, water, acetonitrile, and methanol. All solvents were heavy metal-free based on the specifications supplied by VWR Prolabo. The carotenoid standards used in high-performance liquid chromatography (HPLC) were provided by Sigma-Aldrich (Madrid, Spain). The microorganism strains to test the antimicrobial activity of the extracts were acquired, characterized, and validated by the Spanish Collection of Type Cultures at the University of Valencia (Spain). They are the bacterial species specified in the UNE-EN-13697:2015+A1 Standard because they represent the pathogens of greatest interest for studying sanitizers in the food industry [*Pseudomonas aeruginosa* (NCIMB 8626/ATCC 15442), *Staphylococcus aureus* (NCIMB 9518/ATCC 6538), *Enterococcus hirae* (NCIMB 6459/ATCC 10541), *Escherichia coli* (NCIMB 8545/ATCC 10536), and *Bacillus subtilis* (NCIMB 8054/ATCC 6633)].

### 3.2. Conventional Extraction

Conventional solvent extraction was performed using methanol. The extraction was performed in a shaker incubator at 30 °C for 24 h. The biomass-to-solvent ratio was 1:100 (g:mL). Cell debris were removed by centrifuging the samples (OHAUS, Frontier 5816R, Parsippany, NJ, USA) at 8000 rpm (9300× *g*) and 15 °C for 5 min. The experiment was performed in triplicate (*n* = 3, ± SD) and the supernatant was pooled and labeled as the crude extract until use. A part of the extract was used to obtain the extraction yield. The extraction yield was determined gravimetrically after drying the sample under a nitrogen stream to eliminate the solvent (24-Hole Nitrogen Evaporators, MD200–2N, Ollital Technology, Fugian, China). The extraction yield for conventional extraction was 12.31% ± 0.02% *w*/*w* on average and was expressed as mg of extract/g of the dry weight of *C. onubensis* (benchmark extraction).

### 3.3. Ultrasonic Green Extraction Design

UAE was performed using a ratio of 1:100 (g:mL) biomass and ethanol as the solvent (green extractant). Experiments were conducted using two different ultrasonic equipment: an ultrasonic bath (Ultrasons H-D 3000866 Selecta, Barcelona, Spain) with a power of 330 W (approximately 1–5 W/cm^2^ of the acoustic power or ultrasound power intensity delivered into a liquid), a frequency of 50 Hz, and temperature control and an ultrasonic probe (VCX 750, Vibra Cell Sonics, Newtown, CT, USA) with a power of 750 W, frequency of 20 kHz, a 1/8” (3 mm) titanium probe (maximum amplitude: 40% equal to 226 W/cm^2^ of the acoustic power), and temperature control (12 °C ± 4 °C). To optimize the operation parameters, different values were tested under two different factorial designs (3 × 2) that will elucidate the effects of 2 factors with 3 levels in 11 runs (plus two central points). For the ultrasonic bath, the factors were extraction time (10–30 min) and temperature (30–70 °C). For the ultrasonic probe, the factors were extraction time (5–15 min) and pulsed duration/interval referring to the “on” time (10 s) and “off” time (30–60 s) of the sonicator. Table 1 and Table 2 present the schematics of the ultrasonic experiments. After the extraction, the mixtures were centrifuged at 8000 rpm (9300× *g*) and 15 °C for 5 min to recover the ethanol extracts rich in valuable compounds. They were stored in the dark at −18 ± 2 °C until subsequent analysis.

### 3.4. Quantification of Carotenoids

The ethanolic ultrasonic extracts were evaporated, resuspended in chromatographic methanol, and filtered (Ø = 0.22 µm filter) together with the conventional extracts (in methanol). Thereafter, they were transferred into a chromatography vial and immediately used to quantify carotenoids via liquid chromatography. HPLC was performed on the Beckman System Gold binary delivery system equipped with a UV–vis photodiode array detector (Beckman Instruments, Fullerton, CA, USA) using a C18 column (150 mm × 4.6 mm i.d., 5 µm, SunFire ^TM^ column; Waters, Milford, MA, USA). The flow rate was maintained at 1 mL/min and injection volume was 40 µL of the algal extracts. Ethyl acetate was used as mobile phase A and acetonitrile/water (9:1 *v*/*v*) was used as mobile phase B. The mobile phase gradient was as follows: 0–16 min, 0–60% solvent A; 16–30 min, 60% A; and 30–35 min, 100% A. The selected carotenoids were detected at a wavelength of 450 nm and by comparing the peak areas obtained from the methanolic *C. onubensis* extracts with those obtained from the injected standards (Sigma-Aldrich, 0–50 ppm, ~*R*^2^ = 0.998). Lutein concentration was referred to as dry biomass or weight of the extract, whereas lutein recovery was calculated using Equation (9):

(9) Lutein recovery (% w/w)=(Wc/Wt)×100

where Wc is the mass of lutein (mg) extracted under the ultrasonic conditions described in this study and Wt is the mass of lutein conventionally extracted (using solvent extraction with an average lutein concentration of 3.24 ± 0.11 mg/g, expressed as mg of lutein/g of the dry weight of *C. onubensis*, benchmark extraction).

### 3.5. Quantification of the Bioactive Extracts Using Gas Chromatography–Mass Spectrometry (GC–MS)

The chemical composition of the optimal and conventional *C. onubensis* extracts was analyzed using GC–MS (Agilent 5977B mass selective detector, Santa Clara, CA, USA) Compound identification was achieved by comparing the mass spectra with the NIST Mass Spectrometry Data Center (MS 2.3 software version) as well as by comparing the retention indices with the literature values. Detection was performed in the electron impact ionization mode (70 eV) under the following conditions: capillary column, HP-5 MS (30 m × 0.25 mm; film thickness 0.25 µm); temperature program, 40 °C (held for 1 min), raised to 300 °C at a rate of 25 °C/min (held for 10 min), and increased to 325 °C at a rate of 10 °C/min (held to 5 min); injector temperature, 250 °C; carrier gas, helium; and flow rate, 1 mL/min.

### 3.6. Antimicrobial Activity Assay

The extracts subjected to the antimicrobial activity assay were selected from the optimal conditions obtained using the statistical software based on desirability function together with conventional extraction (in methanol). The ultrasonic bath conditions were 70 °C for 22 min, whereas for the ultrasonic probe, the factors were an extraction time of 12 min and pulsed duration/interval of 10/60 s of the sonicator. The biomass-to-solvent ratio was the same (1:100). The ethanolic and methanolic extracts were evaporated under nitrogen flow and resuspended in dimethyl sulfoxide (DMSO) for the antimicrobial assay. The microorganisms selected were the Gram-negative bacteria *Pseudomonas aeruginosa* and *Escherichia coli* and the Gram-positive bacteria *Staphylococcus aureus*, *Enterococcus hirae*, and *Bacillus subtilis*. The biocidal effects of the extracts were determined using the serial dilution method in 96-well microplates, as described by Navarro et al. [8] and Wiegand et al. [40]. The effectiveness of each biocide against a specific microorganism was tested in triplicate by performing successive dilutions of 50% of the previous well using an automated multichannel pipette. The final volume of each microwell was 200 µL, with 5 × 10^5^ colony-forming units of the tested microorganism. The minimum inhibitory concentration (MIC), which is the lowest concentration of the antimicrobial agent that completely inhibits the visible growth of a microorganism after incubation in the test medium, was calculated. The MIC of each extract was determined by visually inspecting the bottom of the well through an enlarged digital imaging system and determining bacterial growth based on the presence of sediment or defined turbidity. A low MIC value corresponds to more efficient antimicrobial activity. The minimum bactericidal concentration (MBC) of each biocide was determined by inoculating agar plates with the content of the highest dilution wells above the MIC and observing no growth after incubation for 48 h at 37 °C.

### 3.7. Statistical Analysis and Multiple Response Optimization

Ultrasonic experimental designs and data analysis were performed using RSM with Statgraphics Centurion XVIII software (StatPoint Technologies, Inc., Warrenton, VA, USA). The effect of each factor and its statistical significance on each of the response variables such as extraction yield and lutein recovery from *C. onubensis* were also analyzed using the standardized Pareto chart. Furthermore, it was used for data elaboration and statistical analysis, with a 95% level of significance. In addition, mathematical models were obtained, and the significances were accepted at a *p*-value of ≤0.05. All measurements were performed in triplicate (*n* = 3). In the factorial design (3 × 2) involving two factors X_1_ and X_2_, the proposed quadratic model (Equation (10)) for each response variable was as follows:

(10) $$Z=\beta_{0}+\beta_{1}X_{1}+\beta_{2}X_{2}+\beta_{12}X_{1}X_{2}+\beta_{11}X_{1}^{2}+\beta_{22}X_{2}^{2}$$

where Z = estimated response, β_0_ = constant, β_1_ and β_2_ = linear coefficients, β_12_ = interaction coefficients between the two factors, and β_11_ and β_22_ = quadratic coefficients.

Multiple response optimization was performed using the desirability function described by Del Castillo et al. [49], which provides an overall objective function starting from fitting equations obtained for each response variable (the total desirability, D). Equation (11) given below, D ranges from 0 to 1, represents the geometric mean of the desirable range of each response (di), which also varies from 0 (undesirable value) to 1 (the most desirable value).

(11) $$D={\left(\prod_{i=1}^{n}{d_{i}}^{r_{i}}\right)}^{\frac{1}{\sum r_{i}}}$$

where r_i_ is the weight assigned by the user for each response variable and d_i_ is the maximization factor of the response variables [50].

## 4. Conclusions

Extraction techniques are a relevant step in microalgae biorefinery because their optimization leads to a better recovery of molecules with biotechnological interest. In the present study, we confirm that UAE is an easy-to-use, rapid, and green technology with improved data for *C. onubensis* compared with solvent extraction. In particular, the ultrasonic bath increased the anti-inflammatory terpenoid lutein recovery up to 34% more than that of solvent extraction. Furthermore, *C. onubensis* extracts exhibited biocidal effects against Gram-positive and Gram-negative bacteria. Nevertheless, only some molecules produced by *C. onubensis* in alcohol-based extracts were identified; according to the literature, the referred molecules exhibit anti-inflammatory properties and can also be responsible for the determined antimicrobial activity. Five terpene biosynthesis-derived molecules (two sterols) were identified: phytol, neophytadiene, lutein, stigmasterol, and campesterol. Based on their terpenoid-derived nature, we suggest that oxidative conditions, including high-light irradiance and UV light, induce the production of terpenoids, addressing their accumulation and the subsequent enhancement of the antimicrobial activity of *C. onubensis* extracts. Therefore, *C. onubensis*, as a microorganism isolated from acidic medium, jointly with the green extraction techniques (UAE) can be a good combination to improve valuable molecules recovery applied in the field of biotechnology as well as in other industries. In addition, eventual extraction efficiency improvement through the selection and optimization of solvent use or a mix of them should be targeted for studies at a larger scale.

## Figures and Tables

**Table 1:** Experimental design to optimize the parameters for ultrasonic bath extraction of *C. onubensis.* The response variables were extraction yield and lutein recovery. Lutein was used as a marker to compare the extraction efficiency of different methods.

Run	T (°C)	Time (min)	Extraction Yield (% *w*/*w*)	Lutein Recovery (% *w*/*w*)
1	−1	−1	13.05	51.81
2	0	−1	21.50	85.17
3	1	−1	27.98	127.81
4	−1	0	21.15	128.51
5	0	0	31.27	108.68
6	1	0	35.15	129.93
7	−1	1	17.82	88.29
8	0	1	26.00	101.53
9	1	1	31.48	134.27
10	0	0	28.45	125.81
11	0	0	29.01	123.20

Standard deviations of the extraction yield result = 0.989558 and lutein recovery = 0.793382. High level (+1): 70 °C and 30 min; low level (−1): 30 °C and 10 min; and central point (0): 50 °C and 20 min.

**Table 2:** Experimental design to optimize the parameters for ultrasonic probe extraction of *C. onubensis.* The response variables were extraction yield and lutein recovery. Lutein was used as a marker to compare the extraction efficiency of different methods.

Run	Pulse * (s/s)	Time (min)	Extraction Yield (% *w*/*w*)	Lutein Recovery (% *w*/*w*)
1	−1	−1	15.43	37.85
2	0	−1	14.57	40.69
3	1	−1	14.98	69.88
4	−1	0	17.17	51.44
5	0	0	15.71	58.94
6	1	0	16.12	97.85
7	−1	1	16.97	65.36
8	0	1	16.78	54.19
9	1	1	17.34	54.93
10	0	0	14.97	60.64
11	0	0	15.24	57.90

* The pulse duration and pulse interval refer to “on” time (equal to 10 s) and “off” time (from 30 to 60 s) of the sonicator. Standard deviation of the extraction yield results = 0.849697 and lutein recovery = 0.817347. High level (+1): 10/60 s/s and 15 min; low level (−1): 10/30 s/s and 5 min; and central point (0): 10/45 s/s and 10 min.

**Table 3:** Antimicrobial activity of the optimal *C. onubensis* extracts obtained using UAE compared with conventional extraction.

Bacteria	Biocidal Effect (µg/mL)				
	Gram-Negative		Gram-Positive		
	*P. aeruginosa*	*E. coli*	*S. aureus*	*E. hirae*	*B. subtilis*
Extraction method					
Conventional	192/96	n.e./n.e.	n.e./n.e.	192/96	192/96
Ultrasonic bath	552/276	552/276	138/69	9/4	9/4
Ultrasonic probe	262/131	262/131	262/131	4/2	131/65

The biocidal effect is described as MBC/MIC referring to minimum bactericide and minimum inhibitory concentration of *C. onubensis* extracts. Positive control with amoxicillin-clavulanic acid resulted in MBC values from 122 to 145 mg/mL for all pathogenic microorganisms tested. Abbreviations: no effect (n.e.).

**Table 4:** Tentative identification of the anti-inflammatory and antimicrobial bioactive compounds present in *C. onubensis* extracts obtained using solvent extraction and UAE.

Bioactive Compound	Retention Time (min)	Molecular Ion (*m*/*z*) M+	Fragments Profile
Neophytadiene	9.444	278	123, 96, 83, 70, 69, 67, 58, 55, 43
Phytol	10.545	296	123, 95, 81, 72, 69, 68, 58, 55, 43, 41
Campesterol	16.875	400	145, 107, 105, 95, 81, 57, 55, 44, 41
Stigmasterol	17.250	440	91, 81, 79, 69, 67, 55, 44, 43, 41

**Table 5:** Major anti-inflammatory and antimicrobial biomolecules present in *C. onubensis* extracts and their biological functions.

Biomolecule	Chemical Structure	Physiological Role	Bioactivity
Phytol	Diterpenoid	Antioxidant biosynthesis precursor	Anti-inflammatory, antimicrobial
Lutein	Xanthophyll	Light absorption and antioxidant activity against ROS	Anti-inflammatory, antimicrobial, antioxidant
Neophytadiene	Diterpene	Cell defense against stress	Anti-inflammatory, antimicrobial, anxiolytic-like, antidepressant-like, anticonvulsant
Campesterol Stigmasterol	Sterol	Cell defense against oxidative stress, cell membrane fluidity regulation	Anti-inflammatory, antimicrobial, antioxidant, Anticancer

The data are collected by [12,43,44].

## Data Availability

All the data are contained within the article and the Supplementary Materials.

## Supplementary Materials

The following supporting information can be downloaded at https://www.mdpi.com/article/10.3390/md21090471/s1. Figure S1: HPLC profile of main carotenoids present in *C. onubensis* (1) neoxanthin, (2) violaxanthin, (3) lutein, (4) zeaxanthin, and (5) β-carotene; Table S1: Regression coefficients and *p*-value for extraction yield, lutein purity, and lutein recovery in their original units and statistics for the fit obtained by multiple linear regression using ultrasonic bath equipment; Table S2: Regression coefficients and *p*-value for extraction yield lutein recovery in their original units and statistics for the fit obtained by multiple linear regression using ultrasonic probe equipment.
