# Supplementary material for: Ultrasound-Based Recovery of Anti-Inflammatory and Antimicrobial Extracts of the Acidophilic Microalga Coccomyxa onubensis
Source: Mar Drugs. 2023 Aug 27;21(9):471. doi: 10.3390/md21090471 (PMC10532798; doi:10.3390/md21090471)
Supplement: Supplementary file 1 [file marinedrugs-21-00471-s001.zip › marinedrugs-2552160-supplementary.pdf]

Supplementary material

Ultrasound-Based Recovery of Anti-Inflammatory and Antimicrobial Extracts of the Acidophilic Microalga *Coccomyxa onubensis*

Mari Carmen Ruiz-Domínguez [1,*], María Robles [2], Lidia Martín [2], Álvaro Beltrán [3], Riccardo Gava [3], María Cuaresma [2], Francisco Navarro [4] and Carlos Vílchez [2]

[1] Laboratorio de Microencapsulación de Compuestos Bioactivos (LAMICBA), Departamento de Ciencias de los Alimentos y Nutrición, Facultad de Ciencias de la Salud, Universidad de Antofagasta, Antofagasta 1240000, Chile
[2] Algal Biotechnology, CIDERTA-RENSMA, Faculty of Experimental Sciences, University of Huelva, 21007 Huelva, Spain; maria.robles@dqcm.uhu.es (M.R.); lidia.martin@dqcm.uhu.es (L.M.); maria.cuaresma@dqcm.uhu.es (M.C.); cvilchez@dqcm.uhu.es (C.V.)
[3] Bioplagen S.L., Av. Castilleja de la Cuesta, 20-22, Bollullos de la Mitación, 41110 Seville, Spain; alvarobeltran@bioplagen.com (Á.B.); riccardogava@bioplagen.com (R.G.)
[4] Cell Alterations by Exogenous Agents, RENSMA and, Department of Integrated Sciences, Faculty of Experimental Sciences, University of Huelva, 21007 Huelva, Spain; fnavarro@uhu.es
* Correspondence: maria.ruiz@uantof.cl; Tel.: +56-552-633-660

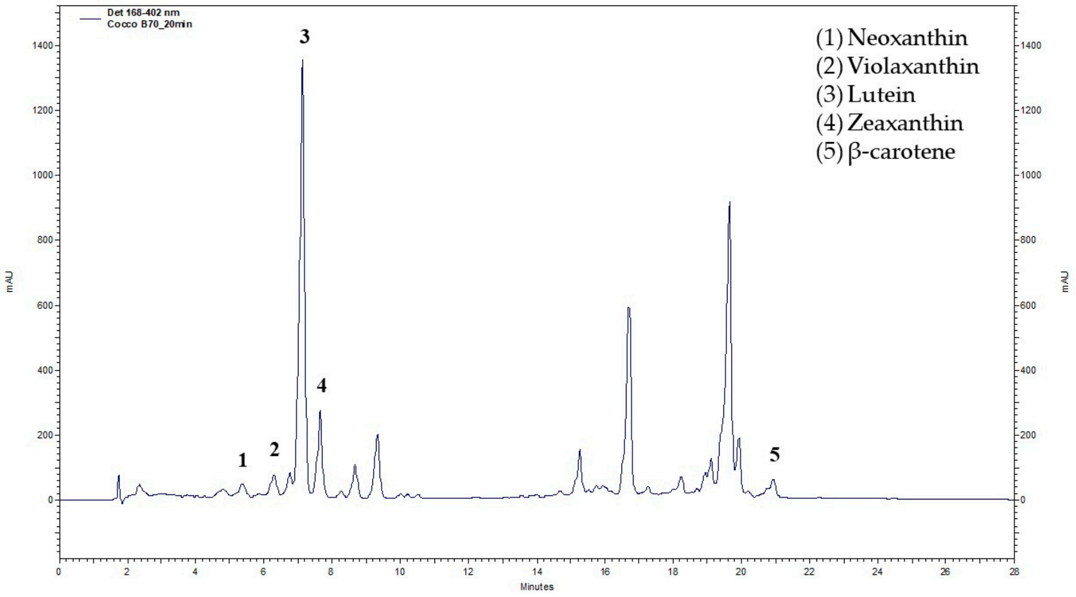

Figure S1. HPLC profile of main carotenoids presents in *C. onubensis*

Table S1. Regression coefficients and p-Value for Extraction yield and lutein recovery in their original units and statistics for the fit obtained by multiple linear regression using ultrasonic bath equipment.

**Terms of the model**	**Extraction yield**		**Lutein Recovery**	
	Estimate	***P-Value***	***Estimate***	***P-Value***
constant	29.5221		119.818	
A: T	7.09833	0.0000*	20.5667	0.0285*
B: t	2.12833	0.0028*	9.88333	0.2030
T × T	-1.29026	0.0845	8.51895	0.4495
T × t	-0.3175	0.5361	-7.505	0.4056
T × t	-5.69026	0.0002*	-27.3511	0.0464*
Statistic for the goodness of fit of the model				
R^2	0.989558		R^2	0.793382
adjusted R^2	0.979115		adjusted R^2	0.586764
RSD	0.370359		RSD	9.39499
P	0.2282		P	0.0471

Note: R^2–determination coefficient, adjusted R^2, RSD–residual standard deviation, P-value of the lack-of-fit test for the model; * - significant coefficients of the model, T–Temperature (ºC), and t – extraction time (min)

Table S2. Regression coefficients and p-Value for extraction yield lutein recovery in their original units and statistics for the fit obtained by multiple linear regression using ultrasonic probe equipment.

**Terms of the model**	**Extraction yield**		**Lutein Recovery**	
	Estimate	***P-Value***	***Estimate***	***P-Value***
constant	15.4821		60.4063	
A: Pi	-0.188333	0.3668	11.335	0.0348*
B: t	1.01833	0.0030*	4.34333	0.3207
Pi × Pi	0.899737	0.0275	12.3692	0.0970
Pi × t	0.205	0.4184	-10.615	0.0792
T × t	-0.0702632	0.8195	-14.8358	0.0582
Statistic for the goodness of fit of the model				
R^2	0.849697		R^2	0.817347
adjusted R^2	0.779571		adjusted R^2	0.634695
RSD	0.26075		RSD	5.13633
P	0.1167		P	0.1087

Note: R^2–determination coefficient, adjusted R^2, RSD–residual standard deviation, P-value of the lack-of-fit test for the model; * - significant coefficients of the model, Pi–Pulse interval (s/s), and t – extraction time (min)
